# Supplementary material for: Light Regulates Secreted Metabolite Production and Antagonistic Activity in Trichoderma
Source: J Fungi (Basel). 2024 Dec 26;11(1):9. doi: 10.3390/jof11010009 (PMC11767173; doi:10.3390/jof11010009)
Supplement: Supplementary file 1 [file jof-11-00009-s001.zip › jof-3328505-supplementary.pdf]

Supplementary materials

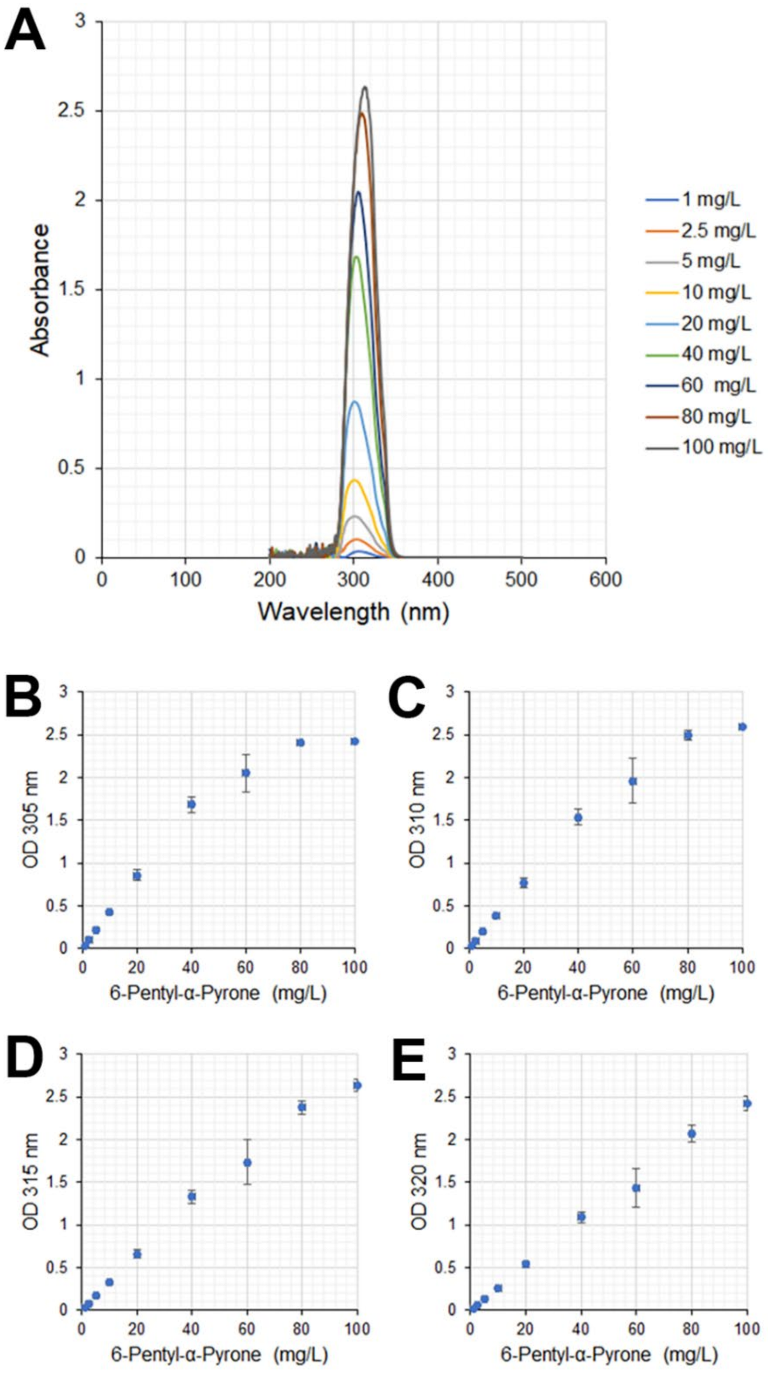

Figure S1. The absorbance of different concentrations of 6-pentyl-α-pyrone dissolved in hexane. **A**, Wavelength scan was carried out from 200 nm to 500 nm with different concentrations of 6-pentyl-α-pyrone dissolved (1-100 mg/L) in hexane. Linearity was analysed at 305 nm (**B**), 310 nm (**C**), 315 nm (**D**), and 320 nm (**E**) wavelengths around the maximum peak detected in **A**, using different concentrations of 6-pentyl-α-pyrone indicated. These results are the average of four replicates.

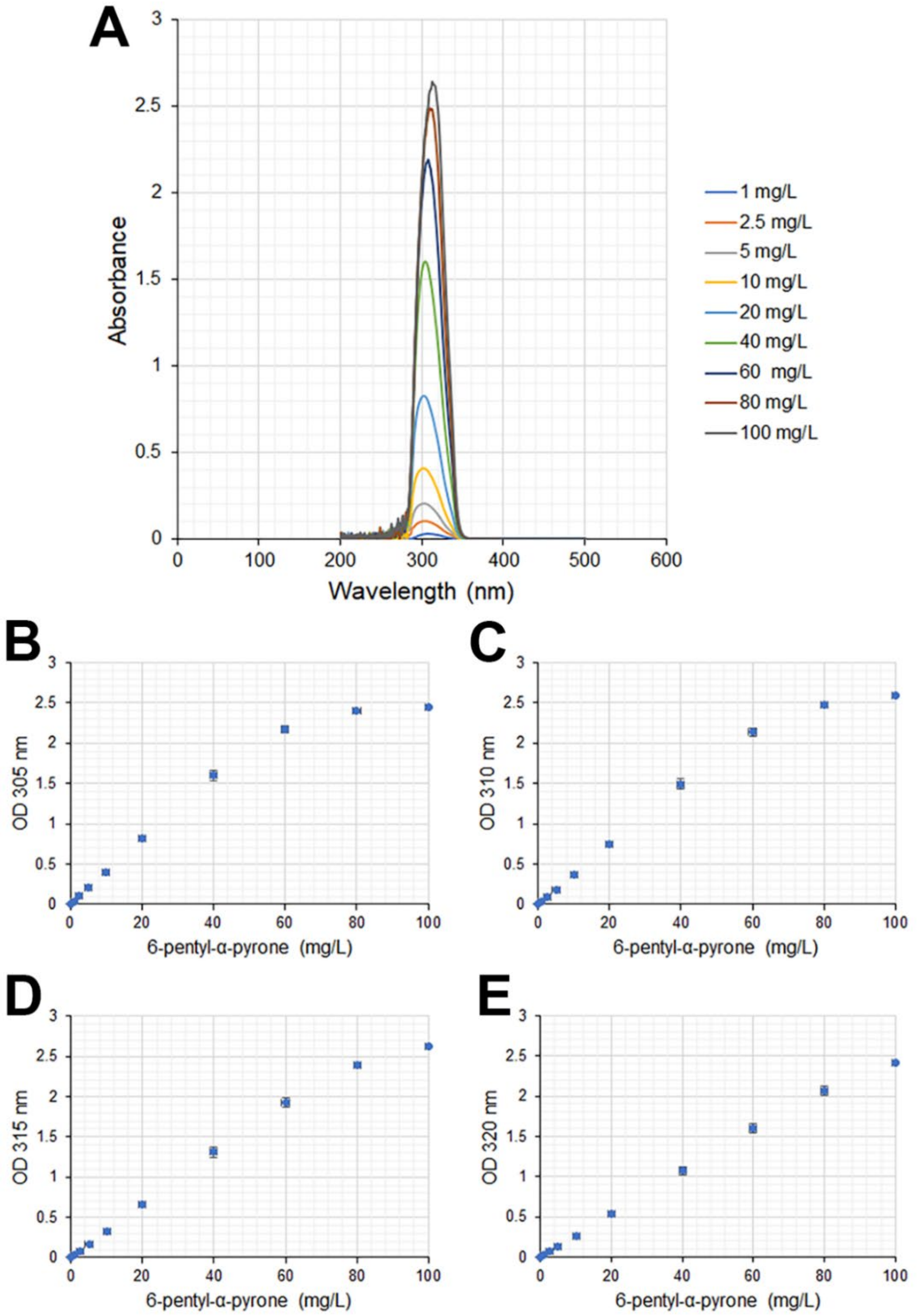

Figure S2. The absorbance of different concentrations of 6-pentyl-α-pyrone dissolved in ethanol. **A**, Wavelength scan was carried out from 200 nm to 500 nm with different concentrations of 6-pentyl-α-pyrone (1-100 mg/L) dissolved in ethanol. Linearity was analysed at 305 nm (**B**), 310 nm (**C**), 315 nm (**D**), and 320 nm (**E**) wavelengths around the maximum peak detected in **A**, using different concentrations of 6-pentyl-α-pyrone indicated. These results are the average of four replicates.

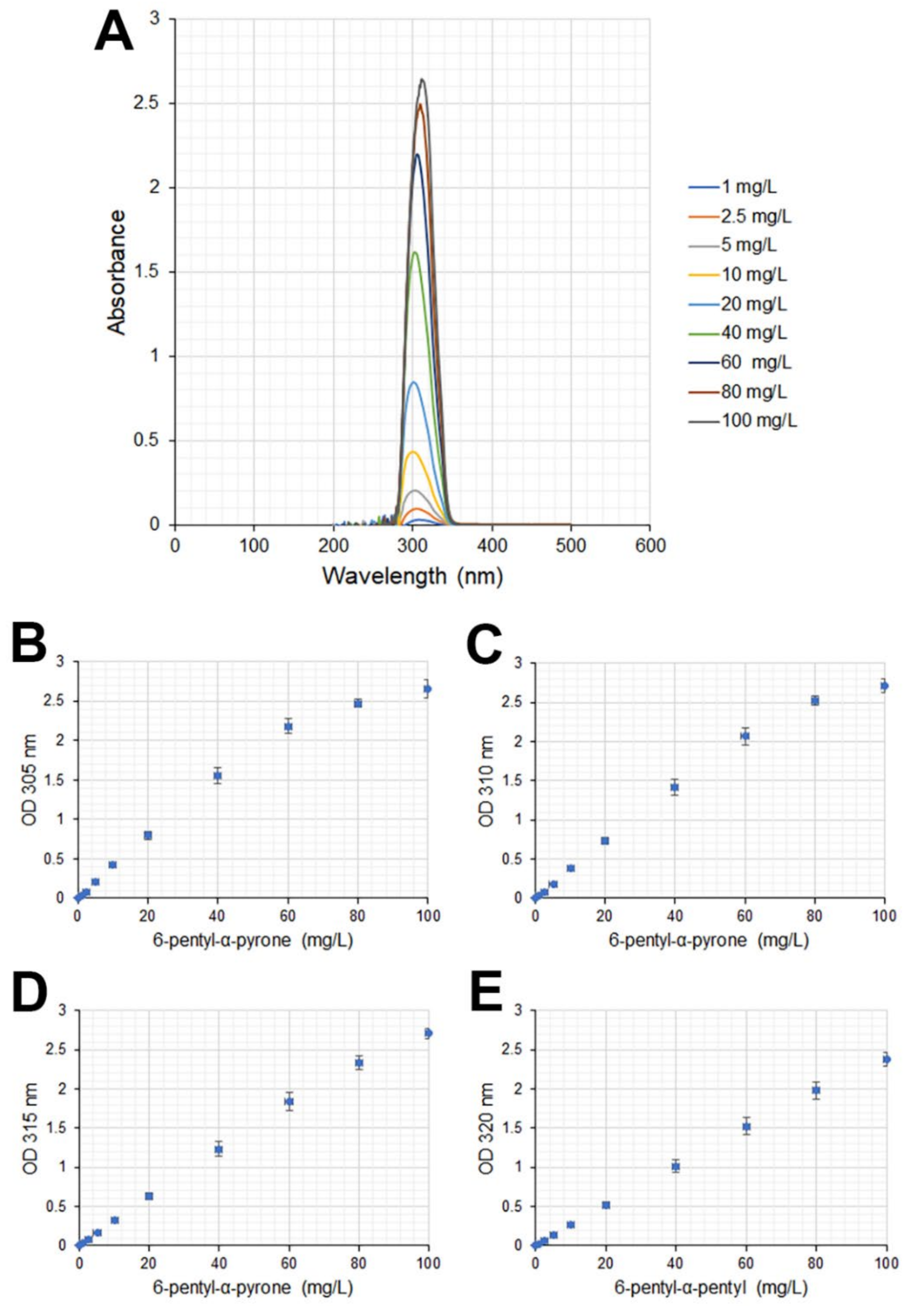

Figure S3. The absorbance of different concentrations of 6-pentyl-α-pyrone dissolved in ethyl acetate. **A**, Wavelength scan was carried out from 200 nm to 500 nm with different concentrations of 6-pentyl-α-pyrone (1-100 mg/L) dissolved in ethyl acetate. Linearity was analysed at 305 nm (**B**), 310 nm (**C**), 315 nm (**D**), and 320 nm (**E**) wavelengths around the maximum peak detected in **A**, using different concentrations of 6-pentyl-α-pyrone indicated. These results are the average of four replicates.

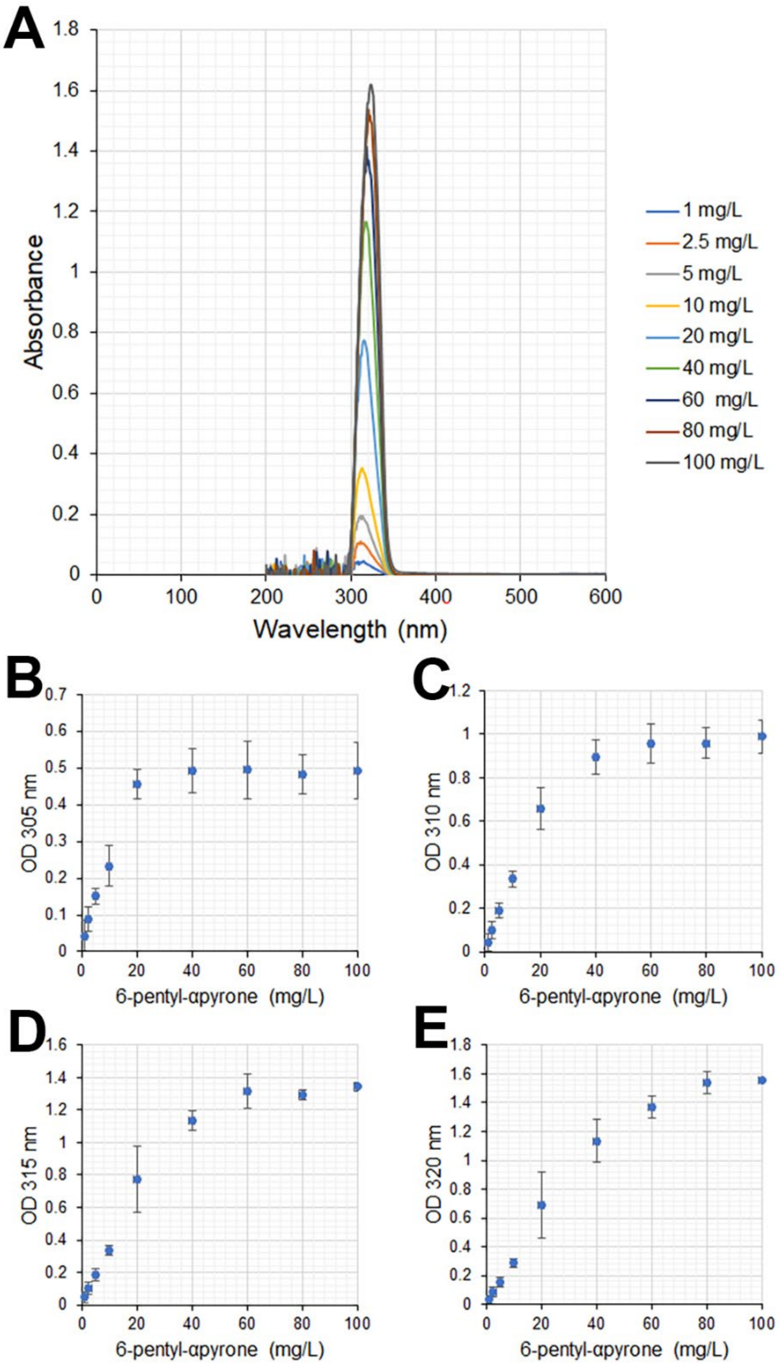

Figure S4. The absorbance of different concentrations of 6-pentyl-α-pyrone dissolved in PDB. **A**, Wavelength scan was carried out from 200 nm to 500 nm with different concentrations of 6-pentyl-α-pyrone (1-100 mg/L) dissolved in PDB. Linearity was analysed at 305 nm (**B**), 310 nm (**C**), 315 nm (**D**), and 320 nm (**E**) wavelengths around the maximum peak detected in **A**, using different concentrations of 6-pentyl-α-pyrone indicated. These results are the average of four replicates.

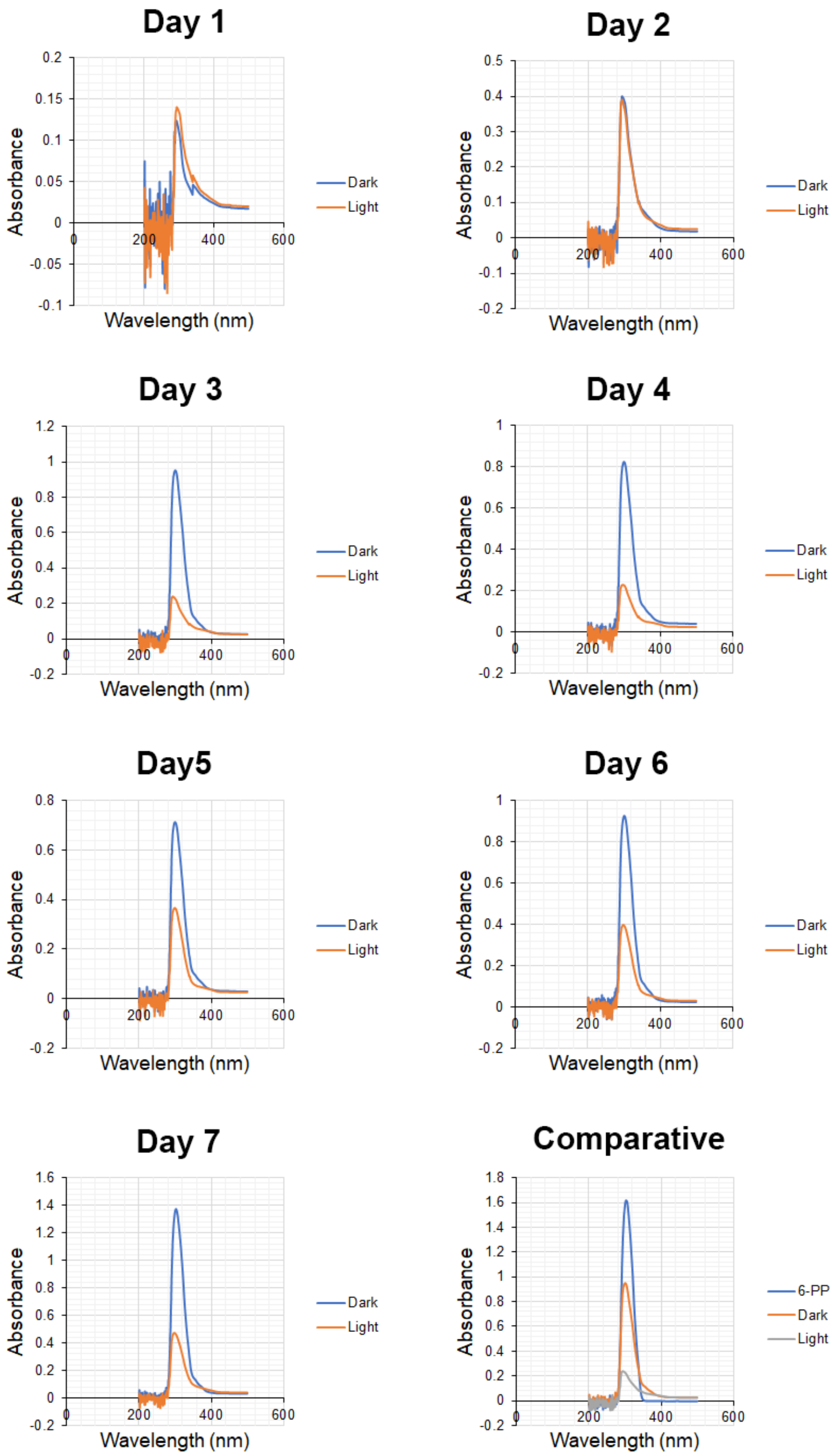

Figure S5. The absorbance spectrum of metabolites extracted produced by *T. atroviride*. An absorbance scan was carried out from metabolites extracted from filtrates of *T. atroviride*, growing in PDB for the days indicated at the top of each graph at 27 °C in dark and light. **Comparative** is comparing the absorbance between metabolites produced by Trichoderma and 6-PP (40 mg/L). These results are the average of three replicates.

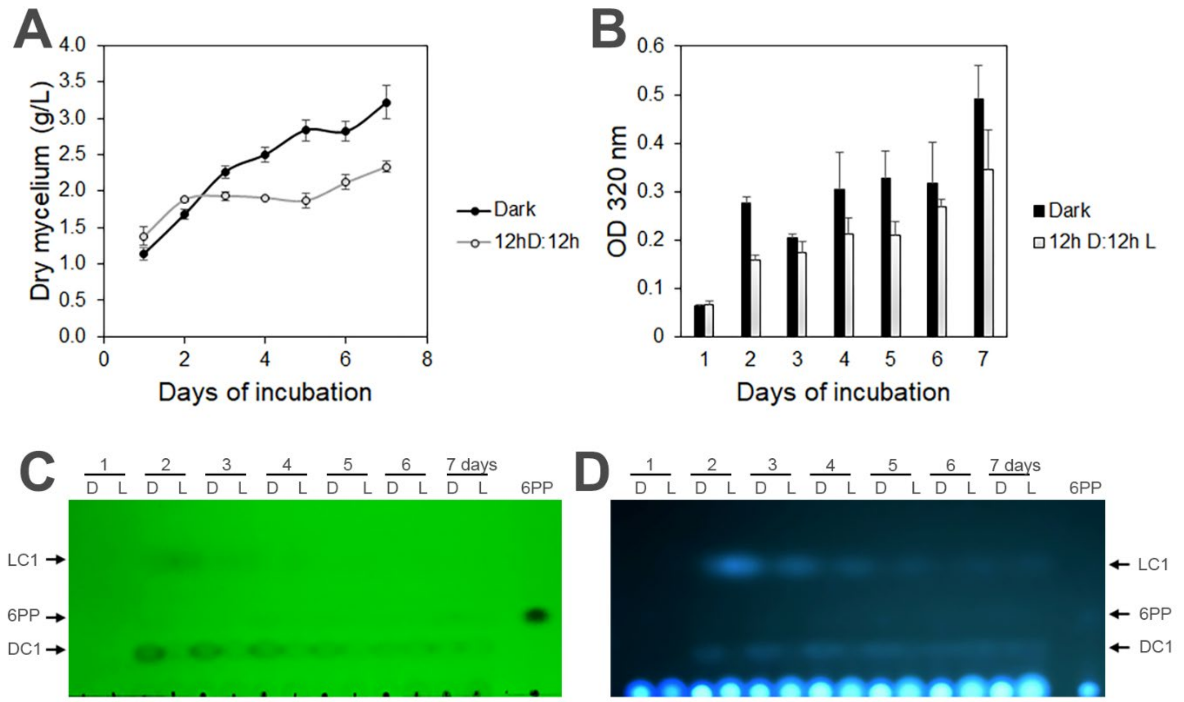

Figure S6. Effect of light regime (dark; 12h dark: 12h light) on *T. atroviride* growth and production of 6-pentyl-α-pyrone. **A**, Growth in PDB over 7 days at 27 °C. (**B**) Absorbance following ethyl acetate extraction from the *T. atroviride* culture. **C-D** Compounds extracted from cultures of *T. atroviride* growing in 12 h dark/12 h light or in the dark treatments for 7 days. The TLCs were exposed to short-wave (**C**) or long-wave (**D**) UV to detect the compounds produced in light (LC) or dark (DC). The vertical bars on each empty or black circle and the bars are (+/-) standard deviation of data from three replicates.

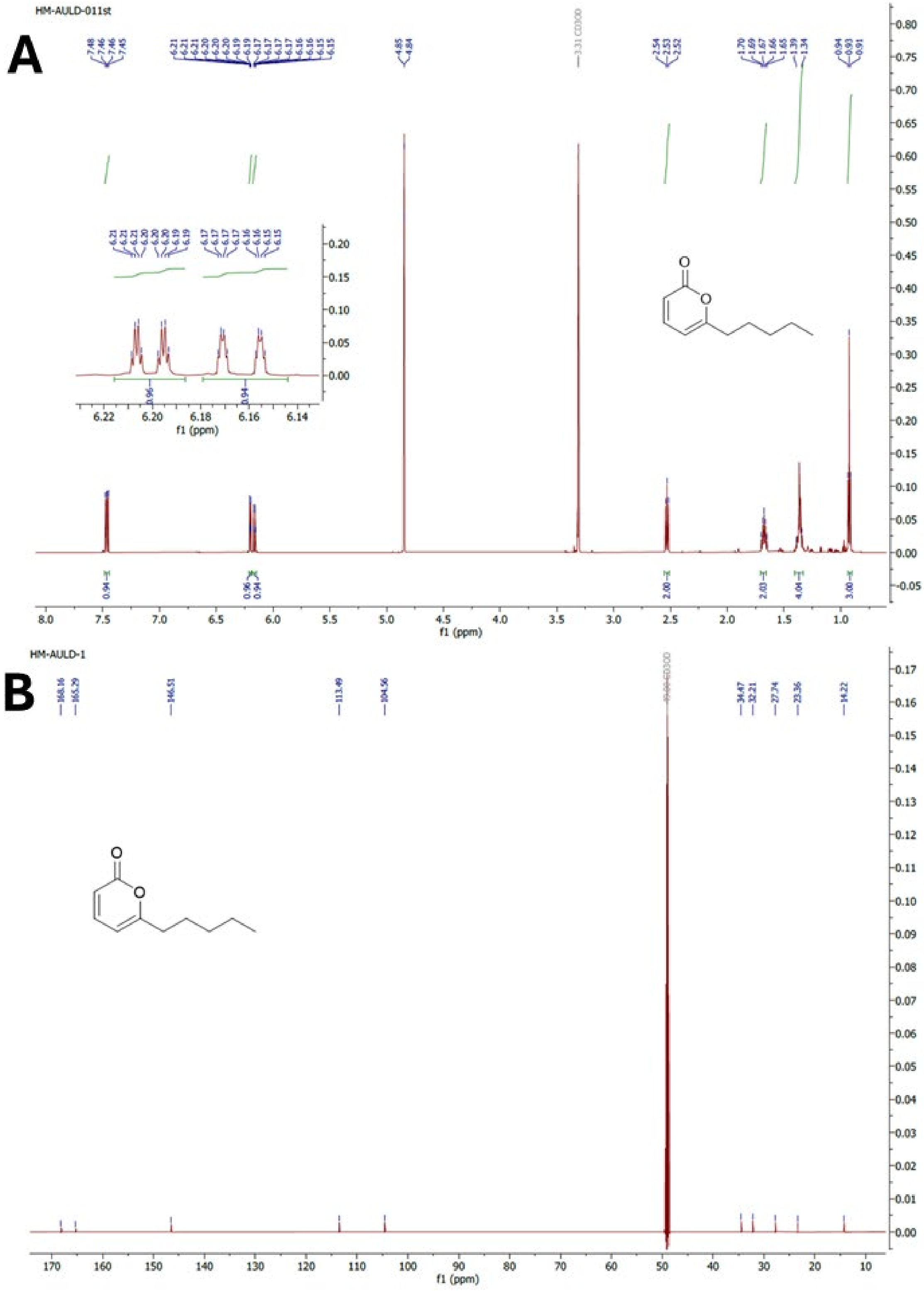

Figure S7. 6-Amyl-α-pyrone isolated from extract NMR spectroscopy. A, ^{1}H NMR spectrum of the molecule (CD_3OD, 600 MHz).**B,** ^{13}C NMR spectrum of the molecule (CD_3OD, 151 MHz).

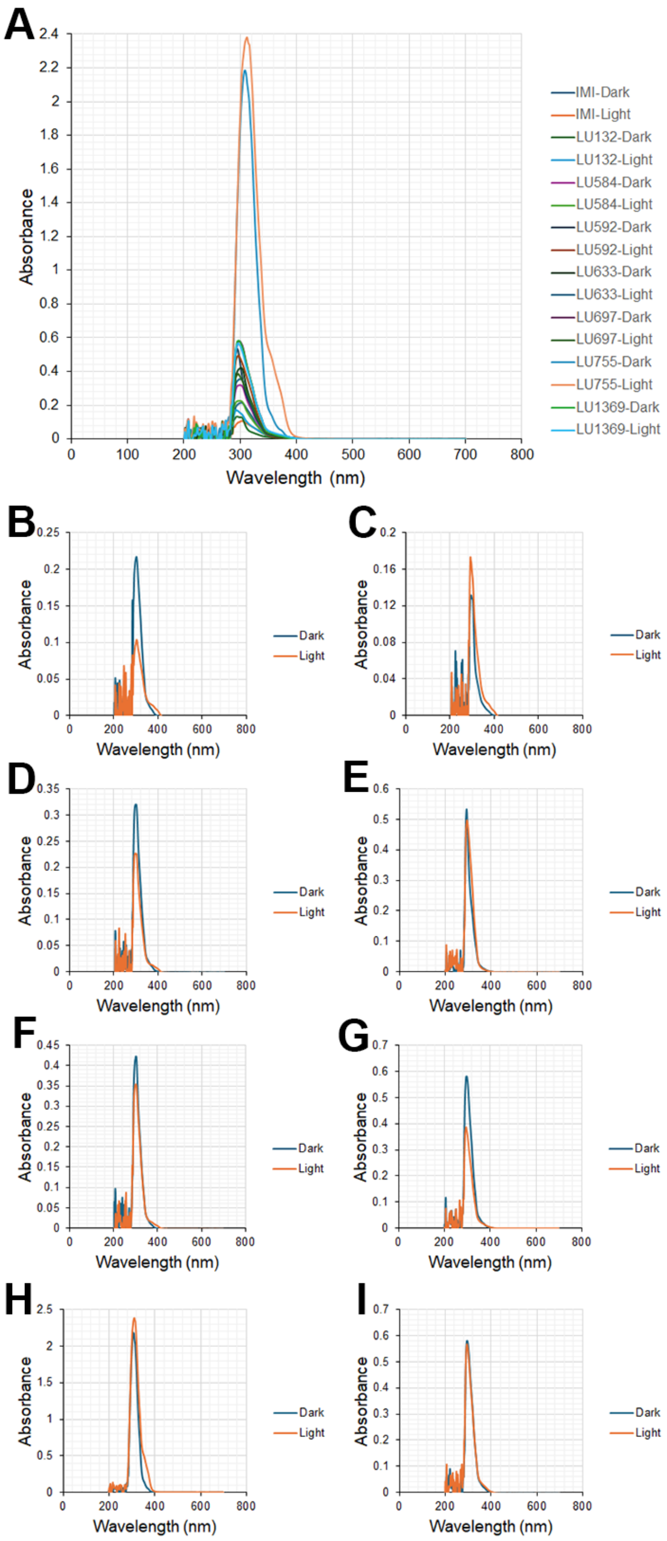

Figure S8. The absorbance spectrum of metabolites secreted by *T. atroviride*. A, the absorbance spectrum was carried out from metabolites extracted from filtrates of different *Trichoderma* strains, growing in PDB for three days at 27 °C in the dark and light. **B**, *T. atroviride* IMI206040; **C**, LU132; **D**, LU584, **E**, LU592; **F**, LU633; **G**, LU697; **H**, LU755; **I**, LU1369. These results are the average of three replicates.

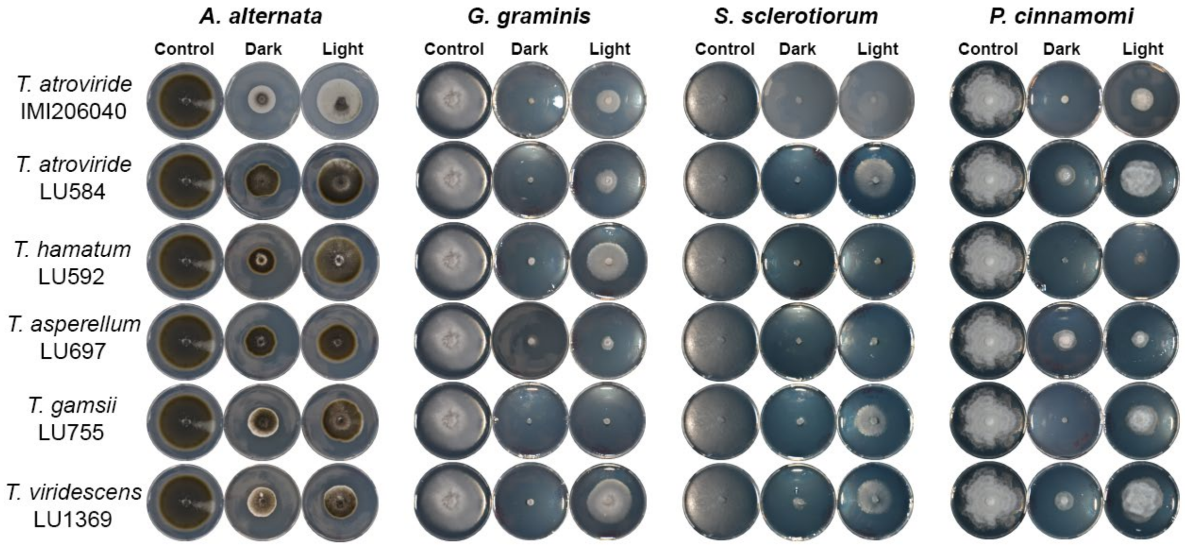

Figure S9. Effect of light on antagonistic activity of *Trichoderma* spp. against plant pathogens. *Trichoderma* was grown on PDA plates covered with cellophane for 48 h at 27 °C, and the cellophane with *Trichoderma* was removed. The indicated plant pathogens were inoculated, and cultures were photographed when the control growth on fresh PDA almost reached the border, two days for *S. sclerotiorum*, five days for *A. alternata* and *G. graminis*, and seven days for *P. cinnamomi*.

Table S1. Average growth inhibition of *Trichoderma* spp. against three fungal and one oomycete pathogens. The colony diameters were measured from colonies grown for two days for *S. sclerotiorum*, five days for *A. alternata* and *G. graminis*, and seven days for *P. cinnamomi*. The experimental variation is represented as the standard deviation in parenthesis.

Trichoderma strain	*A. alternata*		*G. graminis*		*S. sclerotiorum*		*P. cinnamomi*	
	Dark	Light	Dark	Light	Dark	Light	Dark	Light
IMI206040	58.7(7.2)	26.7(5.0)	100.0 (0)	65.5(5.8)	100.0(0)	62.5(10.6)	100.0 (0)	62.2(5.1)
LU584	33.4(3.0)	13.0(1.5)	89.6(0.8)	59.8(4.7)	100.0(0)	47.0(13.2)	67.6(7.7)	29.2(1.1)
LU592	52.0(3.9)	15.0(3.4)	87.5(0.5)	29.8(13.0)	100.0(0)	100.0(0)	100.0(0)	75.4(2.0)
LU697	38.9(1.7)	26.5(1.5)	66.1(11.0)	63.2(8.8)	100.0(0)	100.0(0)	56.2(3.0)	68.5(1.2)
LU755	46.8(1.1)	27.0(6.5)	100.0(0)	87.3(5.0)	85.6(2.4)	53.0(5.1)	100.0(0)	47.7(8.5)
LU1369	42.3(6.5)	35.3(3.7)	88.7(1.1)	39.8(14.4)	83.4(12.9)	49.8(7.0)	61.7(3.7)	23.9(8.3)
